# Development of a nomogram predicting metastatic disease and the assessment of NCCN, AUA and EAU guideline recommendations for bone imaging in prostate cancer patients

**DOI:** 10.1007/s00345-020-03363-0

**Published:** 2020-07-20

**Authors:** Ming-Wei Ma, Xian-Shu Gao, Feng Lyu, Xiao-Bin Gu, Huan Yin, Hong-Zhen Li, Xiao-Ying Li, Xin Qi, Yun Bai, Jia-Yan Chen

**Affiliations:** 1 Department of Radiation Oncology, Peking University First Hospital, No. 7 Xishiku Street, Beijing, 100034 People’s Republic of China; 2 Department of Radiation Oncology, Zhengzhou University First Affiliated Hospital, Zhengzhou, China; 3 Department of Health Science & Technology Strategy Information, Chinese Academy of Medical Sciences & Peking Union Medical College Institute of Medical Information, Beijing, China

**Keywords:** Prostate cancer, Metastasis, Bone imaging, Nomogram

## Abstract

**Purpose:**

We identified the risk predictors related to prostate cancer (PCa) metastasis using contemporary data in a community setting. Then, we assessed the performance of indications for bone imaging recommended from the NCCN, AUA and EAU guidelines.

**Methods:**

Using the Surveillance, Epidemiology, and End Results database (2010–2015), we collected clinicopathological information from PCa patients. The associated risk factors found by multivariate analyses were used to establish forest plots and nomograms for distant metastasis (DM) and bone(s)-only metastasis (BM). We next evaluated the NCCN, AUA and EAU guidelines indications for the discovery of certain subgroups of patients who should receive bone imaging.

**Results:**

A total of 120,136 patients were eligible for analysis, of which 96.7% had no metastasis. The odds ratios of positive DM and BM results were 13.90 times and 15.87 times higher in patients with a histologic grade group (GG) 5 than in the reference group. The concordance index of the nomograms based on race, age, T/N stage, PSA, GG, percentage of positive scores for predicting DM and BM was 0.942 and 0.928, respectively. Performance of the NCCN, AUA and EAU guidelines was high and relatively similar in terms of sensitivity (93.2–96.9%) and negative predictive value (99.8–99.9%). NCCN guidelines had the highest accuracy, specificity and positive likelihood ratio, while negative likelihood ratio was lowest in AUA guideline.

**Conclusion:**

Histologic GG 5 was the foremost factor for DM and BM. NCCN-based recommendations may be more rational in clinical practice. Nomograms predicting metastasis demonstrate high accuracy.

**Electronic supplementary material:**

The online version of this article (10.1007/s00345-020-03363-0) contains supplementary material, which is available to authorized users.

## Introduction

Survival is relatively high for prostate cancer (PCa) among common cancers [1, 2]. Furthermore, the death rate has significantly decreased from its peak since 1993. The latest data showed that the five-year relative survival rates for localized and regional PCa were both higher than 99% [2]. However, only 31% of patients diagnosed at a distant stage survive more than 5 years [2]. As PCa alone accounts for more than 1 in 5 new diagnoses in men in the USA, it remained one of the leading causes of cancer-related death among men in America in 2017 [2] and eighth among both sexes worldwide in 2018 [3]. In addition, it has recently been reported that the decline in PCa mortality has stabilized, and there has been an increased burden of distant stage disease after a decline in prostate-specific antigen (PSA) testing [4]. Since metastasis-free survival is a strong surrogate for overall survival (OS) in PCa patients [5], it is of great significance that we pay close attention to the occurrence of metastasis in PCa patients.

To help identify patients at risk of early metastasis, it is necessary to identify certain unfavorable characteristics associated with the occurrence of metastasis upon diagnosis. It is even helpful in patients with these unfavorable characteristics and without metastasis, since additional follow-up or aggressive treatment to prevent metastasis can be taken into account.

In addition, it is known that bone is the major site of PCa metastasis. Existing clinical guidelines (i.e., guidelines from National Comprehensive Cancer Network (NCCN) [6], American Urological Association (AUA) [7, 8] and European Association of Urology (EAU) [9]) are clear about omitting bone imaging in men with low-risk cancers. However, there is no consensus on the recommendation of imaging for men with high-risk localized cancers. In addition, to the best of our knowledge, the latest recommendations of the guidelines have not been validated in a large sample size.

In this study, we analyzed the risk factors related to PCa metastasis and their correlation degree using contemporary data from the Surveillance, Epidemiology, and End Results (SEER) database. In addition, nomograms that could be used to predict metastasis over the natural course of the disease were established using these factors and may, to some extent, provide references to further improve testing or treatment strategies. We also assessed recommendations to perform a bone imaging by the NCCN, AUA and EAU guidelines [6–9].

## Materials and methods

### Participants and study design

The clinicopathological information of PCa patients in our study was collected from January 1, 2010, to December 31, 2015, from the SEER database. The inclusion criteria consisted of the following: (1) patients with prostate adenocarcinoma and (2) patients who had complete clinical and pathological data. Patients with less than 6 biopsy cores or ambiguous information were excluded. The procedure of the data screening of this study is shown in supplementary Table 1.

**Table 1:** Demographics of men diagnosed with PCa

	All patients (*n* = 120,136) No. (%)	Patients without metastasis (*n* = 116,224) No. (%)^a^ (%)^b^	Patients with metastasis (*n* = 3912) No. (%)^c^ (%)^d^	*P* value
Age, mean ± SD	65.0 ± 8.4	64.8 ± 8.3	68.6 ± 10.3	< 0.001
Median (range)	65 (33–120)	65 (33–120)	68 (34–97)	
Race				0.012
White	94,103 (78.3)	91,137 (78.4)/(96.8)	2966 (75.8)/(3.2)	
Black	19,655 (16.4)	18,942 (16.3)/(96.4)	713 (18.2)/(3.6)	
Asian or Pacific Islander	5889 (4.9)	5669 (4.9)/(96.3)	220 (5.6)/(3.7)	
American Indian/Alaska Native	489 (0.4)	476 (0.4)/(97.3)	13 (0.3)/(2.7)	
Marital status at diagnosis				< 0.001
Married	89,420 (74.4)	86,924 (74.8)/(97.2)	2496 (63.8)/(2.8)	
Other	30,716 (25.6)	29,300 (25.2)/(95.4)	1416 (36.2)/(4.6)	
Insurance status				< 0.001
Insured/any medicaid	118,316 (98.5)	114,542 (98.6)/(96.8)	3774 (96.5)/(3.2)	
Uninsured	1820 (1.5)	1682 (1.4)/(92.4)	138 (3.5)/(7.6)	
PSA level (ng/ml)				< 0.001
0–4	14,062 (11.7)	13,961 (12.0)/(99.3)	101 (2.6)/(0.7)	
4.1–10	74,444 (62.0)	74,021 (63.7)/(99.4)	423 (10.8)/(0.6)	
10.1–20	18,948 (15.8)	18,437 (15.9)/(97.3)	511 (13.1)/(0.7)	
> 20	12,682 (10.6)	9805 (8.4)/(77.3)	2877 (73.5)/(22.7)	
Biopsy GS				< 0.001
≤ 6	46,302 (38.5)	46,206 (39.8)/(99.8)	96 (2.5)/(0.2)	
3 + 4	33,740 (28.1)	33,504 (28.8)/(99.3)	236 (6.0)/(0.7)	
4 + 3	16,357 (13.6)	15,985 (13.8)/(97.7)	372 (9.5)/(0.3)	
8	12,853 (10.7)	11,896 (10.2)/(94.5)	957 (24.5)/(7.4)	
9–10	10,884 (9.1)	8633 (7.4)/(79.3)	2251 (57.5)/(20.7)	
Clinical T stage (AJCC-TNM 2010)				< 0.001
T1	51,763 (43.1)	50,243 (43.2)/(97.1)	1520 (38.9)/(2.9)	
T2	51,973 (43.3)	50,473 (43.4)/(97.1)	1500 (38.3)/(2.9)	
T3a	9834 (8.2)	9632 (8.3)/(97.9)	202 (5.2)/(2.1)	
T3b	5678 (4.7)	5390 (4.6)/(94.9)	288 (7.4)/(5.1)	
T4	888 (0.7)	486 (0.4)/(54.7)	402 (10.3)/(45.3)	
Clinical N stage (AJCC-TNM 2010)				< 0.001
N0	115,930 (96.5)	113,316 (97.5)/(97.7)	2614 (66.8)/(2.3)	
N1	4206 (3.5)	2908 (2.5)/(69.1)	1298 (33.2)/(30.9)	
Proportion of positive cores				< 0.001
< 1/3	56,235 (46.8)	55,968 (48.2)/(99.5)	267 (6.8)/(0.5)	
1/3–2/3	40,007 (33.3)	39,271 (33.8)/(98.2)	736 (18.8)/(1.8)	
≥ 2/3	23,894 (19.9)	20,985 (18.1)/(87.8)	2909 (74.4)/(12.2)	

*SD* standard deviation, *PSA* prostate-specific antigen, *AJCC* American Joint Committee on Cancer, *TNM* tumor node metastasis

^a^Percentage of patients without metastasis with specific character to patients without metastasis

^b^Percentage of patients without metastasis with specific character to all patients

^c^Percentage of patients with metastasis with specific character to patients with metastasis

^d^Percentage of patients with metastasis with specific character to all patients

### Primary outcome and selection of risk factors

According to the 7th edition of the American Joint Committee on Cancer Staging manual, the primary outcome variable for this analysis was the occurrence of distant metastasis (DM), which included nonregional lymph node(s) metastasis (defined as M1a stage), bone(s) metastasis (M1b stage), other site(s) with or without bone metastasis (M1c stage) and bone(s)-only metastasis (BM).

The risk factors for selection were routinely available clinical and pathological variables: age at diagnosis (< 65 or ≥ 65 years), race (white, black or other), marital status, insurance status, PSA level (0–9.9, 10–19.9 and ≥ 20 ng/ml), T stage (T1, T2, T3a, T3b and T4), N stage (N0, N1), percentage of cores containing cancer (defined as the number of positive cores over the total number of cores biopsied, divided as < 1/3, 1/3–2/3 and > 2/3) and biopsy Gleason score (GS) (divided as the histologic grade group (GG) 1, 2, 3, 4 and 5).

### Assessment of the NCCN, AUA and EAU guideline recommendations

We next evaluated the NCCN (Version 1.2020), AUA/ASTRO/SUO (2018) and EAU-EANM-ESTRO-ESUR-SIOG Guidelines (2020) guideline indications for the discovery of certain subgroups of patients who should receive bone imaging. The NCCN guideline recommends bone imaging if the tumor stage is clinical T2b-c and the PSA level is > 10 ng/ml, if the tumor stage is clinical T3 or T4 or the PSA level is > 20 ng/ml, if the GG ≥ 4 or if the primary Gleason pattern is 5 [6]. A bone scan is recommended by the AUA if patients are stratified as more advanced than those in the unfavorable intermediate-risk group. Unfavorable risk is defined as GG 2 (with either clinical stage T2b-c or a PSA level of 10–20 ng/ml) or GG 3 (with a PSA level < 20 ng/ml) [7, 8]. According to the EAU guideline, a bone scan is recommended if patients harbor intermediate-risk disease and GG ≥ 3, high-risk disease, or locally advanced disease, in which intermediate risk is defined as a PSA level of 10–20 ng/ml, GG 2–3 or cT2b [9]. Among these guidelines, there is a consensus that any patient with symptoms consistent with bone metastases should receive imaging, which is not available for analysis. In this section of analysis, we included only patients with bone metastases (M1b) or without metastases (M0). As the classification of cT2 is needed, patients with cT2NOS disease were excluded. cT2NOS means no other information on the sub-classification (T2a, T2b and T2c) for clinical extension except T2. Patients with lymph node metastasis were also excluded.

### Statistical analyses and nomogram construction

We first compared the clinical and pathological variables of patients with or without metastatic disease. Differences in distributions for categorical variables were compared between these 2 groups using the Chi square test and Student’s *t* test. Next, we performed univariate and multivariate analyses to assess the association between metastasis and the variables. The associated risk factors identified by the multivariate analysis through logistic models were used to establish forest plots and nomograms for DM and BM using the R packages “forestplot” and “rms,” respectively. Nomogram performance was assessed using the concordance index (C-index) calibration curve based on the calculated multivariable logistic model. Bootstrapping with 1000 resamples was used for model assessment. Additionally, receiver operating characteristic (ROC) curves were calculated for the regression analysis, nomogram models and constituent variables.

The specific characteristics used to examine the performance of each guideline recommendations were as follows: sensitivity, specificity, positive predictive value (PPV), negative predictive value (NPV), number needed to image (NNI) and overall accuracy. We also calculated the positive likelihood ratio (PLR) and negative likelihood ratio (NLR).

The open-source statistical software R (R Development Core Team 2008, Vienna, Austria) was used to perform statistical analysis. All statistical testing was 2-sided, with a significance level of 0.05.

## Results

### Patient characteristics

Table 1 presents the clinical characteristics of the 120,136 patients eligible for analysis, of which 116,224 (96.7%) had no metastasis and 302 (0.3%), 3077 (2.6%) and 533 (0.4%) were in stages M1a, M1b and M1c, respectively. The median patient age was 65 years for all patients. Patients with DM were slightly older (68.6 ± 10.3 vs. 64.8 ± 8.3, *p* < 0.001), more frequently had a PSA level higher than 20 ng/ml (73.5 vs. 8.4%, *p* < 0.001), more frequently harbored biopsy GG 5 (57.5 vs. 7.4%, *p* < 0.001), had a higher proportion of positive cores > 2/3 (74.4 vs. 18.1%, *p* < 0.001) and more frequently harbored advanced and regional tumor stages (all *p* < 0.05) than those without DM. Moreover, patients without DM tended to have a higher proportion of whites and married and insured individuals.

### Factors associated with DM and BM

Results obtained from the univariate analyses of the relationship between clinical and pathological parameters and DM or BM are shown in supplementary Table 2. All variables incorporated into the univariate logistic regression analysis, except for T3 stage (*p* = 0.09 for DM and 0.67 for BM), were significant predictors of DM and BM (all *p* < 0.05). Therefore, we stratified T stage into T1–3 vs. T4.

**Table 2:** Recommendations for bone imaging according to the NCCN, AUA and EAU PCa guidelines

Overall population *n* = 104,480			M1b stage according to the SEER database					
			M1b *n* = 1651 M0 *n* = 102,829					
Bone imaging recommended			TP			FP		
NCCN	AUA	EAU	NCCN	AUA	EAU	NCCN	AUA	EAU
34,315	53,383	60,757	1539	1600	1591	32,776	51,783	59,166

Bone imaging not recommended			FN			TN		
NCCN	AUA	EAU	NCCN	AUA	EAU	NCCN	AUA	EAU
70,165	51,097	43,723	112	51	60	70,053	51,046	43,663

*SEER* surveillance, epidemiology, and end results, *NCCN* national comprehensive cancer network, *AUA* American urological association, *EAU* European association of urology, *TP* true positive, *FP* false positive, *FN* false negative, *TN* true negative

**Fig. 1:**
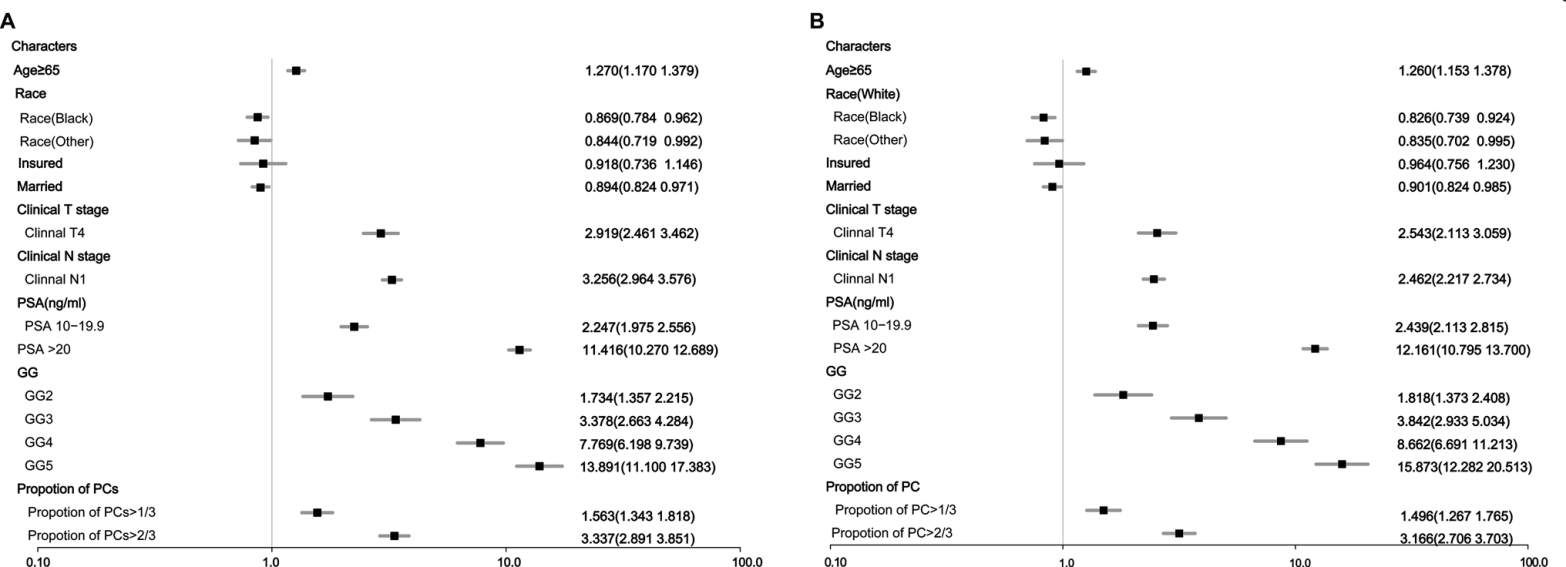
Forest plot of the risk factors for DM (**a**) and BM (**b**)

Figure 1 summarizes the forest plots from multivariate analyses. In multivariate analyses, all the factors mentioned above except for insurance status were significant predictors of both DM and BM (both *p* values < 0.05). To illustrate this point, the adjusted odds ratios (ORs) for a GG 4 were 7.77 times (95% CI 6.20–9.74) higher in patients with DM and 8.66 times (95% CI 6.69–11.21) higher in patients with BM than in patients with a GG 1, respectively, while for patients with a GG 5, the ORs were 13.90 times (95% CI 11.10–17.38) and 15.87 times (95% CI 12.28–20.51) higher in patients with positive DM and BM than in patients in the reference group, respectively. A PSA level higher than 20 ng/ml also accounted for one of the most important risk factors, with ORs of 11.42 (95% CI 10.27–12.69) and 12.16 (95% CI 10.80–13.70) for DM and BM, respectively, compared with a PSA level < 10 ng/ml. Other risk factors included adjacent structure invasion, regional metastasis and the proportion of positive biopsy cores, as well as an uninsured status and a not married status.

Interestingly, the risk factor race (subdivided into white race, black race and other nonwhite races, resulting from univariate analyses) was a protective factor after multivariate analyses. We assessed the proportion of black and other nonwhite patients harboring risk factors, all risk factors except for GG 5 (8.37% for black and 9.03% for nonwhite, *p* = 0.003) and age ≥ 65 years (40.11% for black vs. 54.78% for nonwhite, *p* < 0.001) were found at higher or equivalent proportions in black patients than in white patients. Regarding other nonwhite races, an unmarried status was the only risk factor that was found in a lower proportion in nonwhites than in whites (17.75% for nonwhite vs. 22.68% for white, *p* < 0.001).

### Nomogram construction and validation

**Fig. 2:**
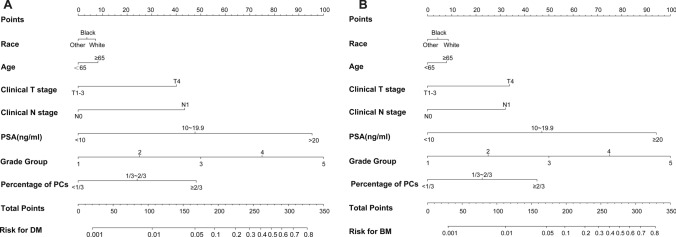
Nomograms predicting DM (**a**) and BM (**b**) for PCa patients

Figure 2 illustrates the nomograms predicted for DM and BM as generated by the factors assessed in the primary cohort. For example, the probability of DM was 45% for an individual white patient aged 68 years old with a PSA level of 30 ng/ml, a cT3a tumor, 9 positive cores demonstrating a tumor after 13 core biopsies from the prostate and a GS of 4 + 5 (GG5). The nomogram for BM may be interpreted in an analogous manner.

**Fig. 3:**
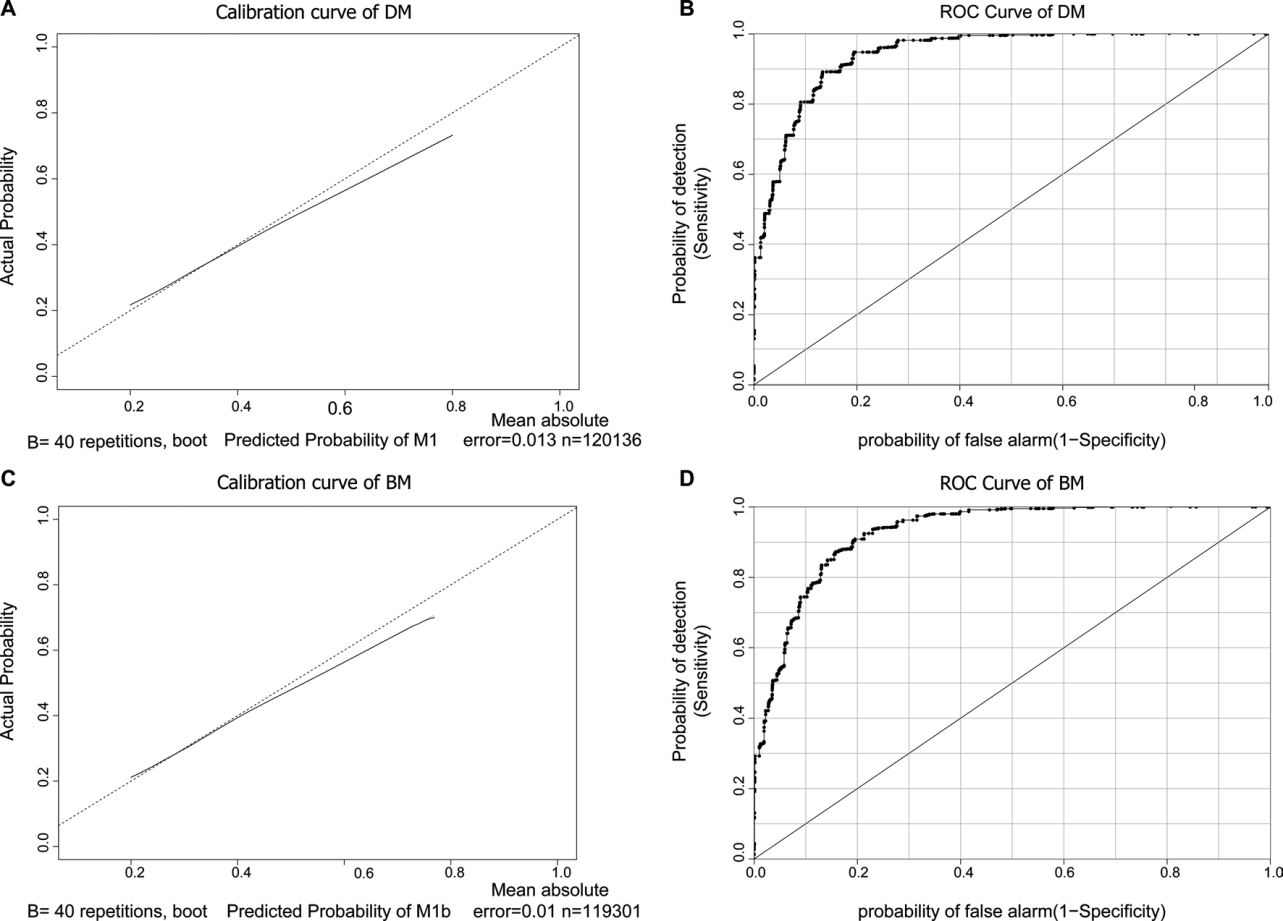
Calibration curves of the nomogram for DM (**a**) and BM (**c**) and ROC curves for DM (**b**) and BM (**d**)

The calibration curves of the predicted probabilities for DM and BM against the observed rates are shown in Fig. 3a, c, with the corresponding ROC curves for DM and BM shown in Fig. 3b, d, respectively. The calibration plots demonstrate a relatively high level of consistency between the predicted and observed probabilities. The C-indexes of the nomograms for predicting DM and BM were 0.942 and 0.928, respectively.

### Assessment of the NCCN, AUA and EAU guideline recommendations for bone imaging

The selection criteria mentioned in Materials and Methods section resulted in 104,480 assessable patients. The ORs of BM according to NCCN, AUA and EAU guideline recommendations prior to treatment were 29.4 (95% CI 24.2–35.6), 30.9 (95% CI 23.4–40.9) and 19.57 (95% CI 15.1–25.3), respectively, in patients in which bone imaging was recommended to those in which bone imaging was not recommended. According to the NCCN, AUA and EAU guidelines, bone imaging was not recommended in 70,165 (67.2%), 51,097 (48.9%) and 43,723 (41.8%) patients, respectively, which indicates that 0.11%, 0.05% and 0.06% of patients harboring BM in the entire cohort, respectively, would have been missed and that 31.37%, 49.56% and 56.63% of patients without BM, respectively, would have been imaged (Table 2).

The performance of the NCCN, AUA and EAU guidelines was high and relatively equivalent in terms of sensitivity and NPV, with maximum differences of 3.7% and 0.1%, respectively. The NCCN guideline had the highest accuracy, specificity and the lowest NNI. Finally, the PLRs and NLRs were 2.92 and 0.10, 1.95 and 0.06, and 1.67 and 0.09, respectively, when applying the NCCN, AUA and EAU guidelines (Table 3).

**Table 3:** Performance characteristics of the NCCN, AUA and EAU PCa guidelines

	Sensitivity^a^, %	Specificity^b^, %	PPV, %	NPV, %	NNI^e^	Accuracy^c^, %	PLR	NLR
NCCN	93.2	68.1	4.5	99.8	22.3	68.5	2.92	0.10
AUA	96.9	50.4	3.0	99.9	33.4	50.4	1.95	0.06
EAU	96.4	42.5	2.6	99.9	38.2	43.3	1.67	0.09

*PLR* positive likelihood ratio = (TP/(TP + FN))/(FP/(FP + TN)), *NLR* negative likelihood ratio = (FN/(TP + FN))/(TN/(FP + TN), *PPV* positive predictive value = TP/(TP + FP), *NPV* negative predictive value = TN/(FN + TN), *NNI* number needed to image = 1/PPV

^a^Sensitivity = TP/(TP + FN)

^b^Specificity = TN/(FP + TN)

^c^Accuracy = (TP + TN)/All

## Discussion

This study used contemporary national surveillance data to investigate, in detail, the correlation between routinely available clinical and pathologic variables and DM or BM in PCa patients and established nomograms that could be used to predict DM and BM over the natural course of the disease using these factors. We found that GG 5 and a serum PSA level > 20 ng/ml were the most principal predictors of both DM and BM, with an increased risk higher than tenfold. Furthermore, the NCCN guideline recommendations for bone imaging may be more rational than those from other organizations.

For PCa patients in which life expectancy is not threatened, early cancer detection can lead to overtreatment. The reduction in PSA testing that began in 2012 [10] may have helped avoid unnecessary impairment in quality of life. However, it also resulted in an increase in metastatic disease, which is the most relevant cause of PCa-related death [4, 5, 11]. Therefore, there is a need to identify the most relevant factors and principles to aid in the identification of early metastasis. Moreover, unnecessary testing in men without metastatic disease may be harmful, as the patients may be exposed to ionizing radiation.

Our investigation is consistent with previous studies showing that high PSA levels and GG are associated with BM [12, 13]. It is obvious that the PSA level is an important predictor since it is generated from cancer cells [14]. By comparison, GG represents the aggressive nature of PCa. GG 5 PCa is aggressive with a poor prognosis [15]. An epidemiologic study showed that the proportion of tumors presenting with a high GG has increased [2]. Even after treatment, multiple studies have shown that GG 5 is the strongest predictor of DM probability [16–19]. The 5-year incidence rates of DM after standard therapy (i.e., radical prostatectomy or radiation therapy) are as high as 24% [19]. There may be a need to apply more aggressive systematic therapy for GG 5 patients before metastasis occurs.

Other studies have demonstrated the correlation between clinical stage and metastasis [12, 20]. Additionally, we identified clinical stage as a risk factor for DM or BM at diagnosis, though for T stage, only T4 was significant in the multivariate setting. The percentage of positive cores is now receiving more attention, as it predicts the presence of lymph node invasion [21] or other advanced disease [22] and is now listed as a risk factor for stratification. In our study, it was also independently correlated with both BM and DM.

Recent studies based on large cohort have demonstrated that the incidence rates or proportions of blacks presenting with metastatic disease were higher than those of whites [11, 23], while some series reported equivalent numbers of whites and blacks [24, 25]. However, multivariable analyses have not shown an association between the black race and metastasis [24]. Interestingly, in our multivariate analyses, the black race had a lower likelihood of metastasis than the white race (OR 0.869 (95% CI 0.784–0.962)) for DM and 0.826 (95% CI 0.739–0.924), for BM, though the proportion of nonwhite patients with metastasis was high (*p* = 0.012). After evaluating the risk factors, we found that the proportions of white patients with GG 5 and age ≥ 65 years were higher than those of black patients, while all the other factors were higher in black patients than in white patients. To some extent, this is further evidence that GG 5 is the crucial risk factor for metastasis. Regarding the effect of race on prognosis, one study including 8820 men with metastatic castration-resistant prostate cancer (mCRPC) found significant prolonged OS in black versus white men, with hazard ratios (HRs) less than 1 for death for black versus white patients [26]. The mechanism of this phenomenon is still under investigation.

As the incidence of metastasis is increasing, more accurate indications are needed to help avoid a missed diagnosis of metastasis. Several studies have validated the available guidelines for bone imaging in different samples of patients [12, 13, 20, 27]. Since the disease staging is shifting and the risk stratification and recommendations for bone imaging have been updated, we assessed the latest indications by the NCCN, AUA and EAU guidelines using current SEER data. We found that all guideline-based recommendations showed high sensitivity (93.2–96.9%) and missed very few patients with BM (0.05–0.11%). Additionally, these recommendations provide a nearly perfect NPV of 99.8–99.9% and a good NLR (0.06–0.10). Among these guidelines, those established by the NCCN demonstrate superior results regarding specificity, accuracy, PPV, NNI and PLR. One of the reason of the superiority of NCCN may be the emphasis on high GG. Thus, considering diagnostic ability and cost-effectiveness, the most instructive indications may be the recommendations from the NCCN guideline. Furthermore, using the independent predictors, we established nomograms for DM and BM, which showed good agreement between predicted and observed probabilities. This model may add some reference value for imaging recommendations to detect metastasis, including BM and DM, which would also be helpful for refining the clinical management of PCa patients. There is no information on the recommendation for DM, yet we may refer to the recommendations for bone imaging, as bone is the most common site for metastasis.

Our study has some limitations. First, the retrospective design and high heterogeneity of used imaging modalities are missing. No information of the exact bone imaging such as bone scan or PSMA PET/CT was provided. Secondly, symptoms such as bone pain are the most essential characteristic for distant disease according to guidelines. There was also no such information in the SEER database. It is preferable if we test the indications in a large cohort of patients in a prospective setting. Second, validation of the indications must be based on confirmed bone metastasis. Even after bone imaging, there may still be few misdiagnoses. Additionally, patients must have a biopsy in order to be risk stratified given the significance of GG, and it is of greater significance if we find any reference to avoid biopsy.

## Conclusions

This analysis was performed to assess the risk factors for metastasis and externally validate the latest clinical guidelines for bone imaging in a population-based cohort of patients. We first identified the foremost predictor, GG 5 for both DM and BM and then found that NCCN-based recommendations may be more rational in clinical practice than other recommendations. In addition, we provide nomograms that can be used to predict metastasis with high accuracy.

## Electronic supplementary material

Below is the link to the electronic supplementary material.

Supplementary material 1 (DOCX 16 kb)

Supplementary material 2 (DOCX 19 kb)
