# Supplementary material for: Development of a nomogram predicting metastatic disease and the assessment of NCCN, AUA and EAU guideline recommendations for bone imaging in prostate cancer patients
Source: World J Urol. 2020 Jul 20;39(6):1815–23. doi: 10.1007/s00345-020-03363-0 (PMC8217023; doi:10.1007/s00345-020-03363-0)
Supplement: Supplementary file 1 — Supplementary material 1 (DOCX 16 kb) [file 345_2020_3363_MOESM1_ESM.docx]

Supplementary table 1. The procedure of the data screening of this study

| Step | Procedure |
| --- | --- |
| 1 | Selection statement of SEER*Stat: {Site and Morphology. Primary Site - labeled} = ‘C61.9-Prostate gland’ AND {Race, Sex. Year Dx. Year of diagnosis}= ‘2010’, ‘2011’, ‘2012’, ‘2013’, ‘2014’, ‘2015’ AND {Stage -7th edition..Derived AJCC T, 7th ed [2010-2015] = ‘T1’, ‘T2’, ‘T2a’, ‘T2b’, ‘T2c’, ‘T2NOS’, ‘T3’, ‘T3a’, ‘T3b’, ‘T3NOS’, ‘T4’ AND {Stage -7th edition.Derived AJCC N, 7th ed [2010-2015] = ‘N0’, ‘N1’ AND {Stage -7th edition.Derived AJCC M, 7th ed [2010-2015]} = ‘M0’, ‘M1’, ‘M1a’, ‘M1b’, ‘M1c’ AND {Site and Morphology. Histologic Type ICD-O-3} = 8140. |
| 2 | Column: Patient ID; Age recode with <1 year olds; Race recode (White, Black, Other); Year of diagnosis; Race recode (W, B, AI, API); Insurance Recode (2007+); Marital status at diagnosis; Derived AJCC Stage Group 7th ed (2010-2015); Derived AJCC T, 7th ed (2010-2015); Derived AJCC N, 7th ed (2010-2015); Derived AJCC M, 7th ed (2010-2015); CS site-specific factor 1 (2004+ varying by schema); CS site-specific factor 3 (2004+ varying by schema); CS site-specific factor 7 (2004+ varying by schema); CS site-specific factor 8 (2004+ varying by schema); CS site-specific factor 9 (2004+ varying by schema); CS site-specific factor 10 (2004+ varying by schema); CS site-specific factor 12 (2004+ varying by schema); CS site-specific factor 13 (2004+ varying by schema) |
| 3 | Patients who do not have complete clinical and pathological data to perform the related analysis were excluded.  Patients with less than 6 biopsy cores or ambiguous information were also excluded. |
