# Supplementary material for: Development of a nomogram predicting metastatic disease and the assessment of NCCN, AUA and EAU guideline recommendations for bone imaging in prostate cancer patients
Source: World J Urol. 2020 Jul 20;39(6):1815–23. doi: 10.1007/s00345-020-03363-0 (PMC8217023; doi:10.1007/s00345-020-03363-0)
Supplement: Supplementary file 2 — Supplementary material 2 (DOCX 19 kb) [file 345_2020_3363_MOESM2_ESM.docx]

Supplementary table 2. Univariate logistic regression analyses for DM and BM

|  | DM^a^ | | BM^b^ | |
| --- | --- | --- | --- | --- |
| Factor | OR (95% CI) | P Value | OR (95% CI) | P Value |
| Age | 1.05 (1.05-1.06) | <0.001 | 1.06 (1.56-1.82) | <0.001 |
| Race |  |  |  |  |
| White | Reference |  | Reference |  |
| Black | 1.16 (1.06-1.26) | <0.001 | 1.11 (1.01-1.22) | 0.04 |
| Other | 1.17 (1.02-1.33) | 0.03 | 1.15 (0.99-1.34) | 0.07 |
| Insurance status |  |  |  |  |
| Uninsured | Reference |  | Reference |  |
| Insured/any Medicaid | 0.40 (0.34-0.48) | <0.001 | 0.43 (0.56-0.65) | <0.001 |
| Marital status at diagnosis |  |  |  |  |
| Other | Reference |  | Reference |  |
| Married | 0.59 (0.56-0.64) | <0.001 | 0.60 (0.56-0.65) | <0.001 |
| PSA level (ng/ml) |  |  |  |  |
| 0~10 | Reference |  | Reference |  |
| 10.1~20 | 4.65 (4.12-5.26) | <0.001 | 4.99 (4.34-5.73) | <0.001 |
| ＞20 | 49.27 (44.78-54.20) | <0.001 | 51.02 (45.77-56.87) | <0.001 |
| Clinical T stage (AJCC-TNM 2010) | |  |  |  |
| T1/2 | Reference |  | Reference |  |
| T3 | 1.09 (0.99-1.20) | 0.09 | 1.02 (0.92-1.14) | 0.67 |
| T4 | 27.59 (24.05-31.64) | <0.001 | 23.73 (20.37-27.64) | <0.001 |
| Clinical N stage (AJCC-TNM 2010) | |  |  |  |
| N0 | Reference |  | Reference |  |
| N1 | 19.35 (17.93-20.88) | <0.001 | 15.34 (14.06-16.73) | <0.001 |
| Biopsy Gleason score |  |  |  |  |
| ≤6 | Reference |  | Reference |  |
| 3+4 | 3.39 (2.67-4.30) | <0.001 | 3.45 (2.62-4.53) | <0.001 |
| 4+3 | 11.20 (8.94-14.03) | <0.001 | 12.16 (9.40-15.74) | <0.001 |
| 8 | 38.72 (31.36-47.81) | <0.001 | 40.46 (31.74-51.57) | <0.001 |
| 9~10 | 125.50 (102.18-154.14) | <0.001 | 131.72 (103.96-166.91) | <0.001 |
| Proportion of positive cores |  |  |  |  |
| <1/3 | Reference |  | Reference |  |
| 1/3~2/3 | 3.93 (3.41-4.52) | <0.001 | 3.79 (3.24-4.43) | <0.001 |
| ≥2/3 | 29.06 (25.61-32.97) | <0.001 | 27.54 (23.96-31.66) | <0.001 |
| ^a^DM=distant metastasis; ^b^BM=bone-only metastasis | | | | |
